# Long-term trends in water chemistry of acid-sensitive Swedish lakes show slow recovery from historic acidification

**DOI:** 10.1007/s13280-014-0563-2

**Published:** 2014-11-15

**Authors:** Martyn N. Futter, Salar Valinia, Stefan Löfgren, Stephan J. Köhler, Jens Fölster

**Affiliations:** Department of Aquatic Sciences and Assessment, Swedish University of Agricultural Sciences (SLU), P.O. Box 7050, 750 07 Uppsala, Sweden

**Keywords:** Acidification, Recovery, Sweden, Base cation decline, Acid neutralizing capacity, Dissolved organic carbon

## Abstract

Long-term (1987–2012) water quality monitoring in 36 acid-sensitive Swedish lakes shows slow recovery from historic acidification. Overall, strong acid anion concentrations declined, primarily as a result of declines in sulfate. Chloride is now the dominant anion in many acid-sensitive lakes. Base cation concentrations have declined less rapidly than strong acid anion concentrations, leading to an increase in charge balance acid neutralizing capacity. In many lakes, modeled organic acidity is now approximately equal to inorganic acidity. The observed trends in water chemistry suggest lakes may not return to reference conditions. Despite declines in acid deposition, many of these lakes are still acidified. Base cation concentrations continue to decline and alkalinity shows only small increases. A changing climate may further delay recovery by increasing dissolved organic carbon concentrations and sea-salt episodes. More intensive forest harvesting may also hamper recovery by reducing the supply of soil base cations.

## Introduction

Acidification has adversely affected many surface waters in Europe, North America, and Asia. Southern Fennoscandia has been especially affected. In Sweden, negative effects of anthropogenic acid deposition on surface waters were first reported in 1967 and as early as 1976, long-term time series were being used to assess the impacts of acid deposition on surface waters (Odén 1976). Acid effects on sensitive species such as Roach (*Rutilus rutilus*) were reported in the 1970s in southwest Sweden (Ålind 1978). In part due to these early studies, European legislation such as the Convention on Long-Range Transboundary Air Pollution (UNECE 2012) led to a >90 % reduction in anthropogenic sulfur deposition in Sweden in comparison to the peak in 1970–1980s (Bertills et al. 2007). This large reduction in sulfur and a smaller reduction in nitrogen deposition should lead to recovery of surface waters from the chronic negative effects of acidification. However, acidification of surface waters is still an ongoing issue in Sweden with concerns about the possibility of re-acidification that may be associated with more intensive forestry (Akselsson et al. 2007).

Henriksen (1979) proposed that loss of alkalinity in surface waters is an indicator of acidification. Alkalinity, or buffering capacity, can be expressed as acid neutralizing capacity (ANC; Reuss and Johnson 1986, see Box 1 for a glossary of acronyms used in the article). Both the Swedish (SEPA) and American (USEPA) environmental protection agencies have proposed five ANC classes related to potential effects of acid deposition on aquatic biota (Table 1). Many countries under the United Nations Economic Commission for Europe (UNECE) umbrella proposed a minimum ANC of 20 µeq L^−1^ to protect ecosystems from harmful effects of acid deposition (cited in Rogora et al. 2013), which is consistent with the Swedish threshold of 20 µeq L^−1^ for waters with no or insignificant buffering capacity. However, adverse biological effects related to anthropogenic acidification can be observed at ANC values much higher than 20 µeq L^−1^ in Swedish surface waters (Fölster et al. 2007). Thus, acidification assessments in Sweden are based on a ΔpH criterion related to effects on fish and benthic invertebrates (Fölster et al. 2007). Lakes are assumed to have acidified if ΔpH has declined by >0.4 pH units compared to a reference condition estimated using the MAGIC model (Moldan et al. 2013). A relative change is used instead of a threshold, since many Swedish lakes are naturally acidic due to high levels of natural organic acids (Erlandsson et al. 2011). It should be noted that the ΔpH method is not entirely consistent with ANC-based assessments of anthropogenic acidification. The use of 20 µeq L^−1^ was based on studies from Norwegian streams, which typically have much lower dissolved organic carbon (DOC) concentrations than Swedish lakes and are thus less affected by organic acidity.

**Box 1:** Explanations of acronyms used in the article

Acronym	Definition
ANC	Acid neutralizing capacity
BCS_ANC	Estimated base cation surplus acid neutralizing capacity (Eq. 3)
CB_ANC	Estimated charge balance acid neutralizing capacity (Eq. 1)
DOC	Dissolved organic carbon
IKEU	Integrerad kalkningseffektuppföljning—liming studies
MAGIC	Model of acidification of groundwater in catchments
OAA_ANC	Estimated organic acid adjusted acid neutralizing capacity (Eq. 2)
SEPA	Swedish Environmental Protection Agency
SQL	Structured Query Language
TOC	Total organic carbon
UNECE	United Nations Economic Commission for Europe
USEPA	United States Environmental Protection Agency
SD	Site density

**Table 1:** Comparison of United States (USEPA) and Swedish (SEPA) environmental protection agency alkalinity classification schemes. The breakpoint indicates the highest alkalinity values at which a classification can be applied. The lowest alkalinity value for a class is indicated by the breakpoint in the row immediately above. For example, the SEPA “Poor buffering capacity” class applies to water bodies with alkalinity values between 50 and 100 µeq L^−1^

Breakpoint (µeq L^−1^)	USEPA	SEPA
0	Acute	
20	Severe	No or insignificant buffering capacity
50	Elevated	Very poor buffering capacity
100	Moderately acidic	Poor buffering capacity
200		Good buffering capacity

Cosby et al. (1985) propose a seven-stage conceptual model of acidification and recovery. Stage 1 “Pre-acidification” is analogous to reference conditions when a water body and its surrounding catchment are assumed to be at steady state prior to any increase in anthropogenic sulfur deposition. In Sweden, 1860 is assumed to be the reference year for pre-acidification conditions (SEPA 2007). In stage 2, effects of acidification start to become apparent as anthropogenic sulfur deposition is adsorbed to catchment soils; strong acid anion and base cation concentrations in soil water rise and alkalinity decreases. In stage 3, high concentrations of anions in the soil begin to deplete the pool of exchangeable base cations. In stage 4, a new steady state is achieved in the soil. Stage 5 starts when deposition returns to levels consistent with stage 1. In stage 6, soil base saturation recovers to pre-acidification levels. During stage 6, soils are still recovering from acidification, and base cation concentrations in soil and surface waters will decline. Stage 7 is the hypothetical return to a pre-acidification state. Most acid-sensitive catchments in Sweden are currently in either stage 5 or 6.

Long-term lake monitoring to assess recovery from acidification has been conducted in Scandinavia (Skjelkvåle et al. 2007), Finland (Vuorenmaa and Forsius 2008), Italy (Rogora et al. 2013), Ireland (Burton and Aherne 2012), Canada (Keller 2009; Clair et al. 2011), the United States (Waller et al. 2012), and the United Kingdom (Monteith et al. 2014). While acid deposition is a significant problem in China (Larssen et al. 2006) and other parts of south Asia (Hicks et al. 2008), long time series showing acidification effects on lake water chemistry are lacking. Monitoring in Europe and North America show that lakes are responding to the decline in acid deposition but recovery of all surface waters is far from complete (Garmo et al. 2014).

A considerable amount of research effort has been devoted to documenting recovery of surface waters from anthropogenic acidification in Sweden. In a study of regional patterns in riverine base cation concentrations, Lucas et al. (2013) showed that streams in southern Sweden responded to declines in acid deposition but that patterns in northern rivers were more closely related to climate variability. Löfgren et al. (2009, 2011) have documented declines in sulfate, ionic strength, and total aluminum as well as increases in pH, ANC, and DOC at ten headwater forest catchments in Sweden. Skjelkvåle et al. (2007) noted that Swedish lakes had shown clear signs of recovery between 1990 and 2004, with increases in pH and ANC and declines in sulfate. Fölster and Wilander (2002) demonstrated a similar pattern of recovery for Swedish streams.

Surveys in parts of south Sweden show that forest soils are still recovering from acidification (Löfgren et al. 2009; Akselsson et al. 2013). The delay in recovery of soils slows the recovery of surface waters. However, there are clear signs of an ongoing recovery from acidification in soil water of mineral soils. Löfgren and Zetterberg (2011) documented declines in sulfate, ionic strength, and total aluminum (Al) as well as increases in pH, ANC, and DOC between 1987 and 2008 at 33–72 % of 68 sampling sites in southern Sweden. Such trends are less common in soil water from northern Sweden (Pihl Karlsson et al. 2011). Moldan et al. (2013) presented a regional model-based analysis of acidification in Swedish lakes. They suggested that while the majority of acidified lakes are recovering, ongoing acidification of forest soils is slowing lake recovery in nearly half of the modeled catchments.

Increases in surface water DOC concentrations associated with declines in acid deposition have been reported in much of the northern hemisphere (Monteith et al. 2007). While the largest increases in DOC have been observed at acidified sites, water bodies that were not significantly acidified have also shown increasing DOC trends consistent with a reduction in acid deposition (Oni et al. 2013). Based on mass-balance estimates, it has been proposed that recovery from acidification may be further delayed if more intensive forest harvesting depletes soil base cation pools (Akselsson et al. 2007). However, long-term empirical data from sites subject to whole-tree harvesting (Zetterberg et al. 2013) and dynamic model simulations (Zetterberg et al. 2014) indicate low impact on base cation leaching to soil solution and stream water, respectively, due to restricted mobile anion leakage.

Here we present an analysis of long-term (1987–2012) trends in water quality parameters for 36 acid-sensitive Swedish lakes. We focus on acidification-related parameters, DOC, and estimates of ANC.

## Materials and methods

### Study sites and determinands

Time series from candidate lakes across Sweden sampled for the Trend, Reference, and IKEU projects were available (see Fig. 1 in Fölster et al. 2014). None of the candidate lakes had been limed and all had been sampled in at least 10 years between 2000 and 2012, either by national monitoring programs or county authorities. All chemical data were obtained from the Swedish national monitoring database maintained by the Department of Aquatic Sciences and Assessment at the Swedish University of Agricultural Sciences (Fölster et al. 2014). All determinands were analyzed using SWEDAC accredited methods. Trends are presented from either 1987 or 1988 for all determinands except measured alkalinity, which starts in 1994. From 1994 onwards alkalinity was determined as the amount of acid or base needed to titrate pH in a sample to pH 5.6 under N_2_ purging, removing CO_2_. Hence, in samples with pH values below 5.6, “alkalinity” represents acidity. Total organic carbon (TOC) was measured as CO_2_ after combustion using a Shimadzu TOC-VPCH analyzer, after acidification and sparging to remove inorganic carbon. Further information about analysis methods, detection limits, etc., are available in Fölster et al. (2014) and in the database web site (http://www.slu.se/vatten-miljo/ visited 13/08/2014)

**Fig. 1:**
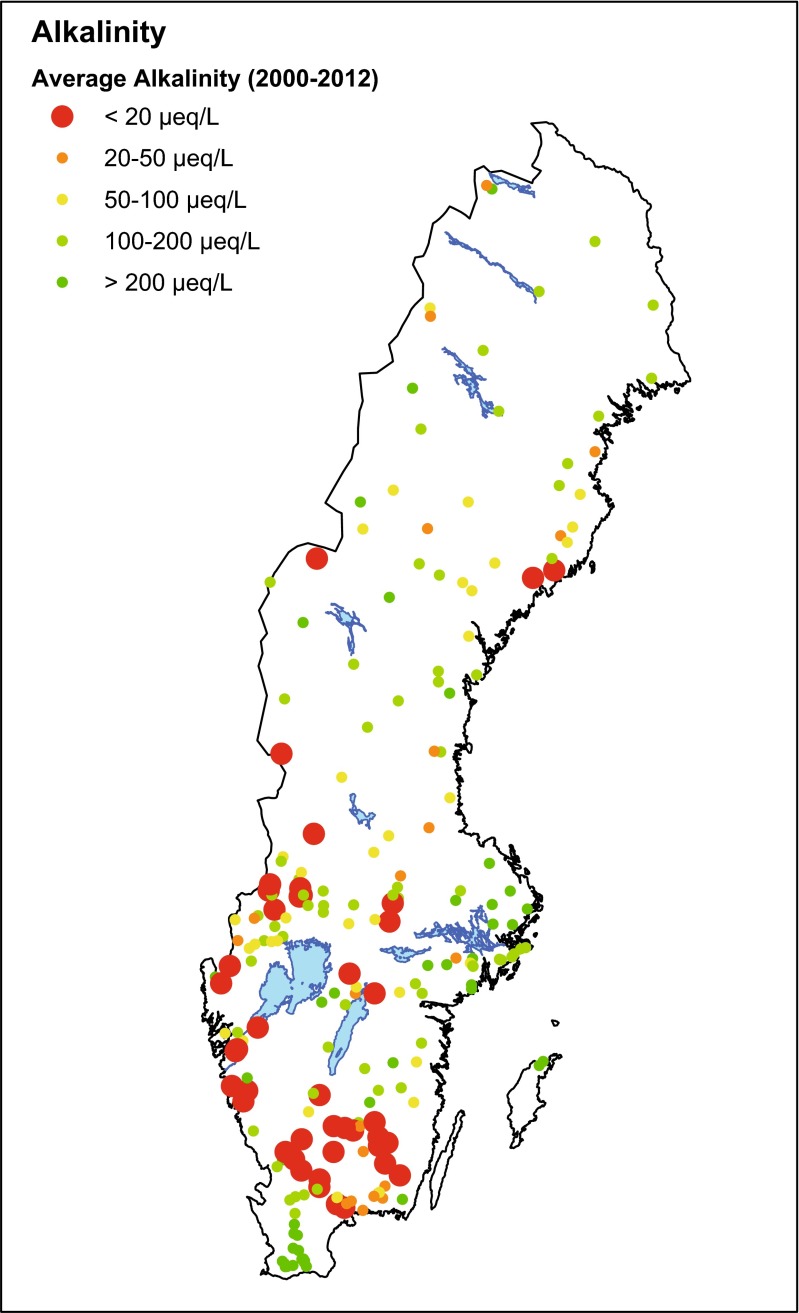
Map of Sweden showing location of lakes for which long-term alkalinity could be estimated (*colored dots*). The 36 lakes with alkalinity <20 µeq L^−1^ were selected for the trend analysis

The analysis presented here is restricted to lakes with long-term (1987–2012) measured concentration time series and low measured alkalinity. Annual average concentration values were estimated by identifying the shallowest depth sampled on each collection date and then calculating annual arithmetic averages from these samples. Average pH was estimated by back-transforming average H^+^ concentrations. Low-alkalinity lakes were identified by calculating the average measured alkalinity, based on at least 10 years of observations between 2000 and 2012. It was possible to estimate long-term average alkalinity for 215 lakes (Fig. 1). These 215 lakes were classified using accepted breakpoints for biota (Table 1). The 36 lakes with long-term average alkalinity below 20 µeq L^−1^ were retained as candidates for further analysis. Almost all of the lakes with no or insignificant buffering capacity (<20 µeq L^−1^), are located either along the west coast or in the south of Sweden (Fig. 1). These regions of Sweden have received the heaviest historical acid deposition loads, primarily from the UK, Germany, and Poland. Additionally, this region receives the largest sea-salt deposition and has the highest forest production in Sweden. Low-alkalinity lakes outside southwest Sweden probably represent a mix of acidified and naturally acidic water bodies.

Time series for individual determinants were included if a lake had been sampled in 20 or more of the 25 years between 1987 and 2012. This constraint resulted in 32–36 lake time series per determinand. Trends were assessed for measured alkalinity, pH, individual and total base cations (Ca^2+^, Mg^2+^, K^+^, and Na^+^), individual and total strong acid anions (SO_4_^2−^, NO_3_^−^, and Cl^−^), total aluminum, and TOC. As most of the TOC in Swedish lakes is present in dissolved form (Gadmar et al. 2002), TOC is assumed to be synonymous with DOC. Trend significance was assessed using a Mann–Kendall test with a Bonferroni correction for multiple comparisons. Trends were also evaluated for a number of calculated parameters related to organic acidity and acid neutralizing capacity. Organic acidity was assessed using a triprotic acid model (Hruška et al. 2003). Trend slopes were calculated using Sen’s slope. All trend, organic acidity, and ANC calculations were performed using SQL in Microsoft Access.

### Estimates of acid neutralizing capacity, organic acidity, and reference condition pH

There are several methods of estimating acid neutralizing capacity (ANC). Charge balance ANC (CB_ANC; µeq L^−1^) is defined as the sum of positive charge associated with base cations minus the sum of negative charge associated with strong acid anions (Reuss and Johnson 1986).

1 $$ {\text{CB\_ANC}} = \left[ {{\text{Ca}}^{{2 + }} } \right] \,+\, \left[ {{\text{Mg}}^{{2 + }} } \right] \,+\, \left[ {{\text{K}}^{ + } } \right] \,+\, \left[ {{\text{Na}}^{ + } } \right] - \left[ {{\text{SO}}_{4}^{{2 - }} } \right]\, -\, \left[ {{\text{NO}}_{3}^{ - } } \right] \,-\, \left[ {{\text{Cl}}^{ - } } \right]. $$

CB_ANC is routinely used in acidification assessments as it is derived from easily measured constituents and is relatively unaffected by pH or partial pressure of CO_2_. However, CB_ANC can be subject to significant measurement error (Evans et al. 2001) and neglects the contribution of organic acids to negative charge. Both Lydersen et al. (2004) and Lawrence et al. (2007) have proposed modifications to CB_ANC to account for the negative charge associated with strong organic acids. Lydersen et al. (2004) propose an organic acid adjusted ANC (OAA_ANC; µeq L^−1^) based on the concentration, site density, and strong acid fraction of DOC:

2 $$ {\text{OAA}}\_{\text{ANC}}={\text{CB}}\_{\text{ANC}} - \left( {1/3} \right)*\left[ {\text{DOC}} \right]*{\text{SD}}, $$

where [DOC] is expressed in mg C L^−1^, a site density (SD) of 10.2 µeq (mg C)^−1^ is used, and it is assumed that 1/3 of the charged sites are permanently dissociated. Site densities and p*K* values in the Lydersen et al. (2004) study were taken from Hruška et al. (2003). Lawrence et al. (2007) developed a similar model, which they refer to as base cation surplus ANC (BCS_ANC; µeq L^−1^) and defined as follows:

3 $$ {\text{BCS\_ANC}} = {\text{CB\_ANC}}-\left( { 0. 0 7 1 *\left[{\text{DOC}} \right] - 2. 1} \right). $$

Organic acidity was estimated as the sum of charge associated with dissociated organic acids estimated using a standard triprotic acid dissociation model.

Here, we used values of 3.8, 4.7, and 5.5 for p*K*a_1_, p*K*a_2_, and p*K*a_3_ respectively, a site density of 7.0 µeq (mg DOC)^−1^, and measured pH. Values were estimated using data from 242 samples collected from 30 lakes during 2011–2012. The average charge balance error in that data set is below 1.5 µeq L^−1^ giving rise to a charge balance error of <1.5 % on average. The average error in prediction of pH using the current model is 0.08 pH units when using charge balance alkalinity in accordance with the earlier results of Köhler et al. (2002), however, it should be noted that the site density used here is somewhat lower than those used in that study (Köhler et al. 2002).

Recovery from acidification was assessed by comparing change in measured pH values from 1990 and 2010 relative to modeled reference pH. Lakes were assumed to have acidified if pH was >0.4 units below reference values (Fölster et al. 2007). Reference (1860) pH values were obtained from the MAGIC model (Cosby et al. 1985, 2001). The calibration process used to estimate anthropogenic acidification since 1860 is described in Moldan et al. (2013). In total, 2903 lakes were modeled across Sweden to estimate the degree of regional acidification in Sweden. A subset of those lakes has been used in this study. Change in pH was estimated based on changes between hind-cast pre-industrial (1860) and present-day ANC. Pre-industrial ANC was calculated by MAGIC and pH was estimated using the following equation where [org^−^] is the sum of organic anions, Al^n+^ is the sum of cationic aluminum species and all terms have units of µeq L^−1^:

4 $$ \left[ {{\text{H}}^{ + } } \right] = \left[ {{\text{CO}}_{3}^{2 - } } \right] + \left[ {{\text{HCO}}_{3}^{ - } } \right] + \left[ {{\text{org}}^{ - } } \right] + \left[ {{\text{Al}}^{n + } } \right] - \left[ {\text{ANC}} \right]. $$

Present-day DOC and the triprotic model of Hruška et al. (2003) are used to estimate [org^−^]. Bicarbonate and carbonate concentrations are estimated assuming 4X oversaturation with respect to atmospheric CO_2_ and concentrations of cationic Al species are assumed to be equal to the average in the calibration data set (Fölster et al. 2007).

To assess changes between reference conditions and contemporary conditions, a conceptual model described by Valinia et al. (2014) was used. The conceptual model makes a graphical presentation of movement toward or away from reference conditions over time. Reference condition values are plotted on the horizontal axis and observed on the vertical. The diagonal 1:1 line shows when reference and present-day conditions are the same. Further diagonal lines can be added to show acceptable deviations from reference conditions. In brief, the conceptual model highlights where remediation measures needed to achieve a return to reference conditions should be prioritized.

## Results

**Fig. 2:**
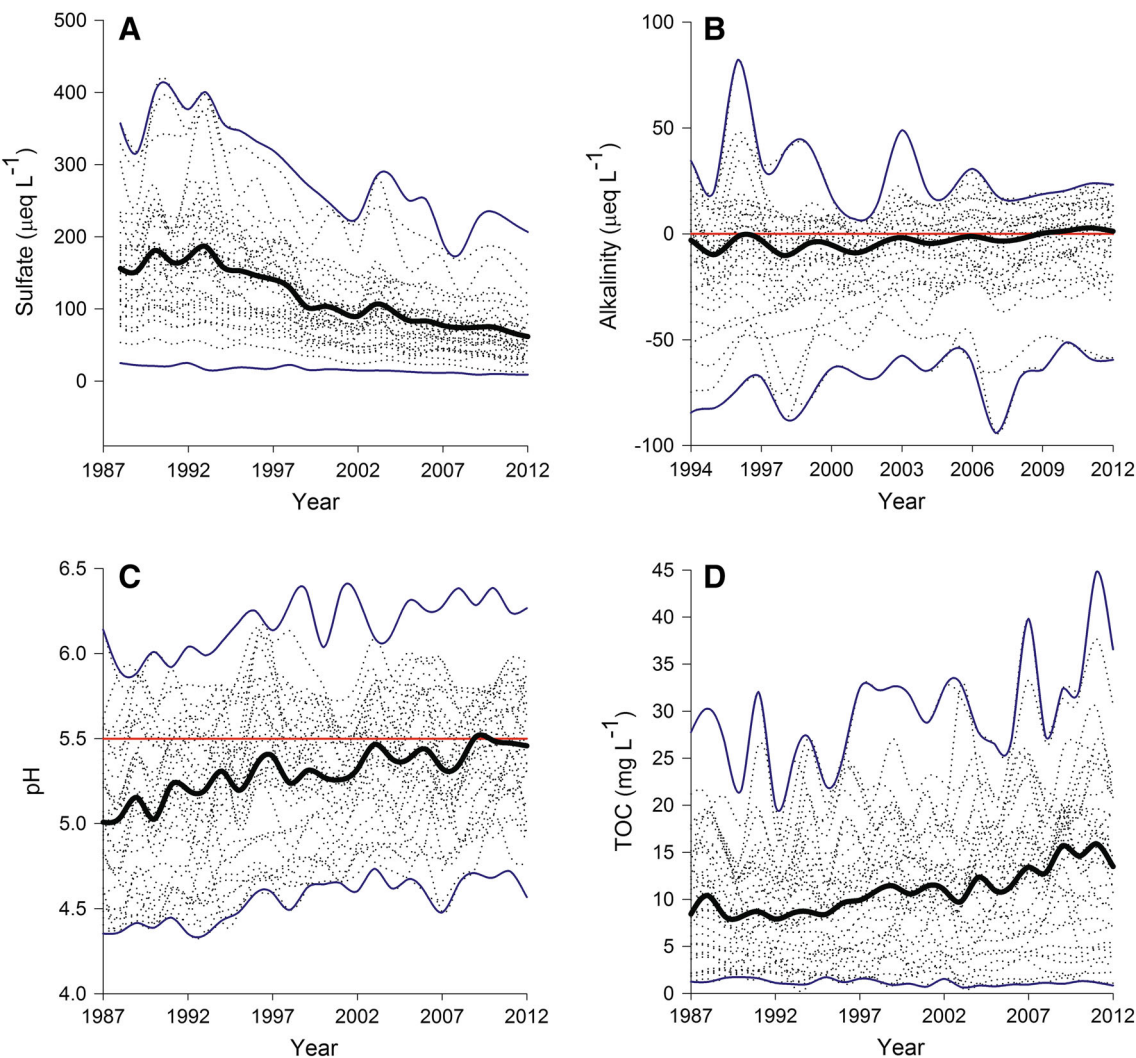
Spaghetti plots of annual sulfate concentration (**a**), alkalinity (**b**), pH (**c**) and total organic carbon (TOC; **d**) concentrations in acid-sensitive Swedish lakes. The *thick black line* shows the median value for observations made in a given year. *Thin blue lines* represent minimum and maximum values reported in a year. The *thin grey lines* represent individual lake time series. The *red* *reference lines* represent an alkalinity of 0 in **b** and a pH of 5.5 in **c**

Overall, the trends in lake chemistry were consistent with a recovery from acidification. Sulfate declined in almost all lakes (Fig. 2a) and many lakes displayed increasing DOC trends. Small increases were also observed for measured alkalinity (Fig. 2b) and pH (Fig. 2c). Total aluminum concentrations remained relatively stable (not shown) and base cation concentrations declined. The overall average pH for all study lakes (*n* = 34) increased by approximately 0.5 pH units between 1987 and 2012 (Fig. 2a). There was a considerable range in pH values, with pH > 6 observed in at least one lake in almost all years. With the exception of 2007, minimum pH had risen above 4.5 in all lakes by 1996. Despite the near ubiquitous increase in pH, there was relatively little change in alkalinity (*n* = 31, Fig. 2b). Since consistent measurements began in 1994, average alkalinity has followed a slight upward trend, which is much smaller than inter-annual variability. Many lakes had no or very low (<0 µeq L^−1^) average alkalinity throughout the whole time period. A small number of lakes had consistently positive alkalinity, and many lakes switched between positive and negative values. Sulfate concentrations declined in all lakes (*n* = 35, Fig. 2c). Overall average concentrations of sulfate declined by approximately 2/3; from 150 µeq L^−1^ in 1988 to 50 µeq L^−1^ in 2012. Declines in sulfate concentrations for individual lakes were approximately proportional to starting concentrations and can be linked to the decline in atmospheric sulfur deposition (Löfgren et al. 2011; Moldan et al. 2013). There was a wide range in DOC concentrations for the study lakes (Fig. 2d) ranging from <2 mg C L^−1^ to >20 mg C L^−1^ (*n* = 33, Fig. 2d). Overall average concentrations increased from 8.5 mg C L^−1^ in 1988 to 14.5 mg C L^−1^ in 2012, with consequent increases in organic acidity. This large increase in average DOC was matched by considerable inter-annual variation in individual lake DOC concentrations.

**Fig. 3:**
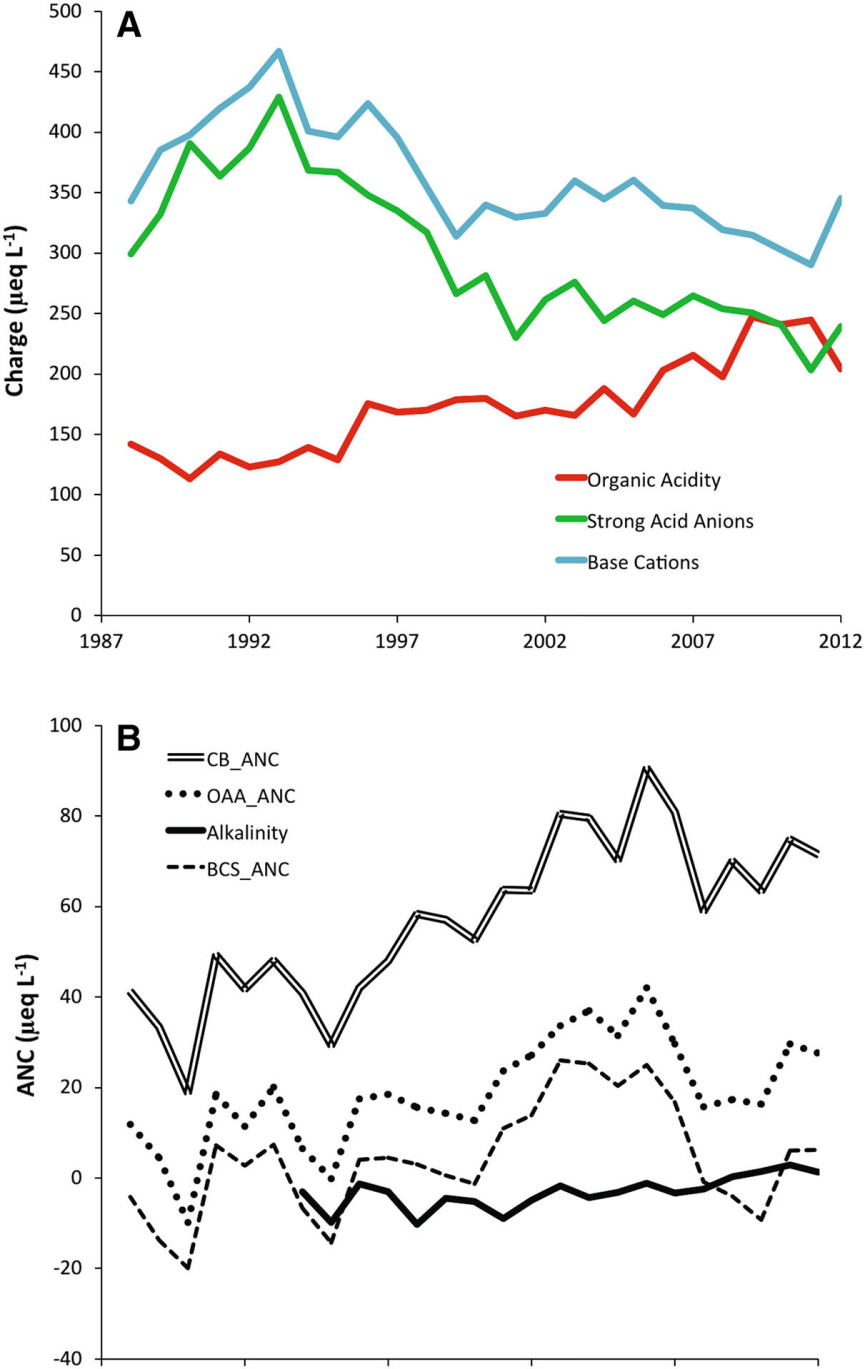
**a** Overall average trends in charge associated with base cations, strong acid anions and organic acids between 1988 and 2012; **b** Overall average trends in measured alkalinity and calculated acid neutralizing capacity (ANC) parameters between 1988 and 2012. Measured alkalinity is presented from 1994 to 2012. CB_ANC is charge balance alkalinity (Eq. 1). OAA_ANC is the organic acid alkalinity proposed by Lydersen et al. (2004, Eq. 2) and BCS_ANC is the base cation surplus ANC of Lawrence et al. (2007, Eq. 3)

Trends in average base cation, strong acid anion, and organic acid concentrations (Fig. 3a) are consistent with a recovery from acidification to stage 5 of the Cosby et al. (1985) conceptual model. Both strong acid anions and base cation concentrations have declined over the period of record. The observed increase in CB_ANC (Fig. 3b) was associated with a faster decline in strong acid anions than that observed for base cations. Modeled charge associated with organic acids has increased due to the increase in DOC concentrations in most lakes. It is noteworthy that by 2012 the overall average charge associated with organic acids was approximately equal to that associated with strong acid anions.

Measured alkalinity and calculated ANC estimates displayed a relatively complex pattern (Fig. 3b). Overall average measured alkalinity displayed a small positive trend over the period of observation. All calculated ANC estimates displayed positive trends and were generally higher than measured alkalinity. Organic acidity adjusted ANC (OAA_ANC and BCS_ANC) were more consistent than CB_ANC with measured alkalinity, but OAA_ANC diverged from measured values in more recent years.

**Fig. 4:**
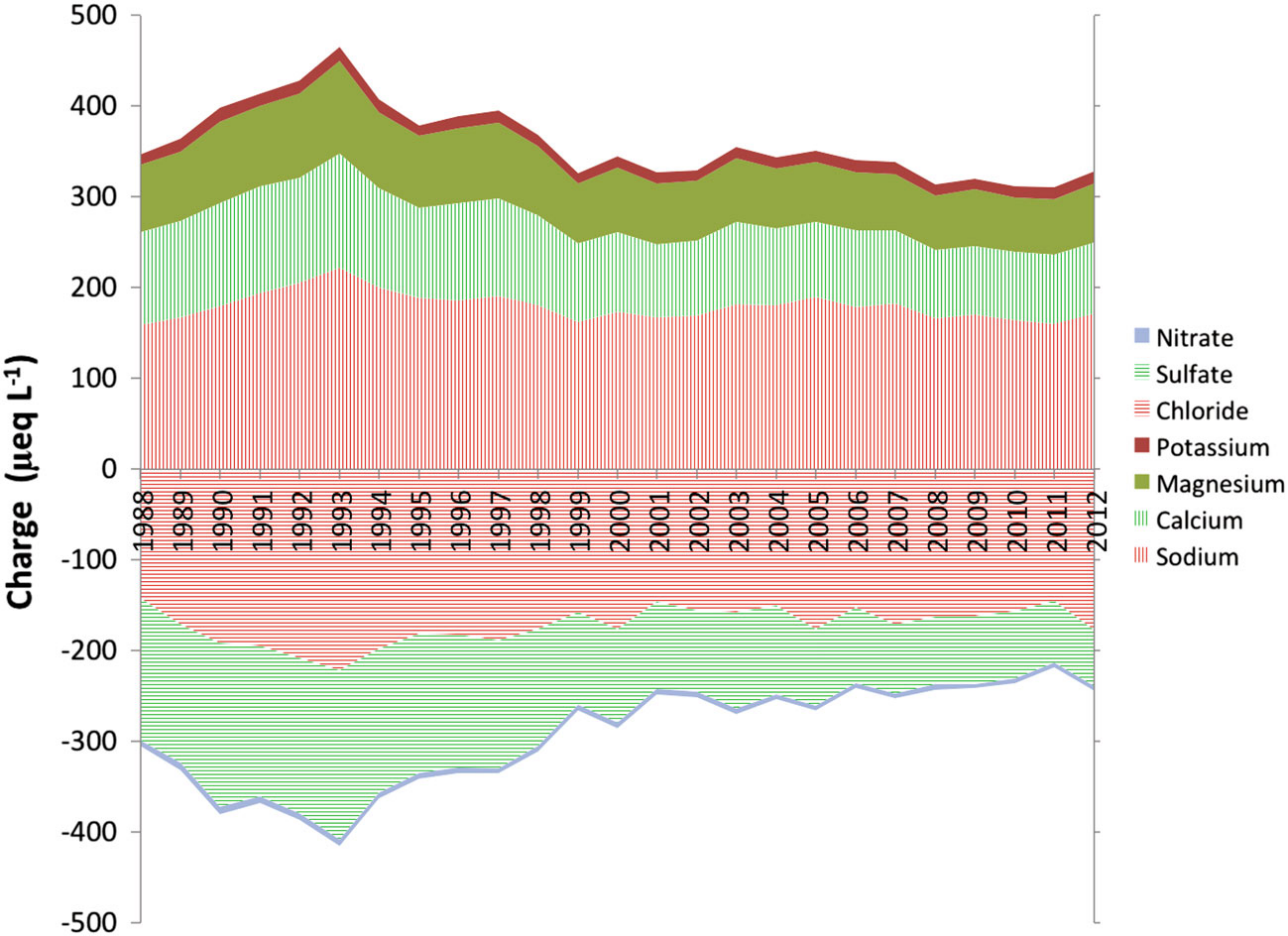
Contribution of individual ions to overall charge balance ANC (Eq. 1) between 1988 and 2012

Analysis of the contribution of individual ions to CB_ANC showed a shift over time (Fig. 4). The negative charge associated with chloride has remained relatively constant while the charge associated with sulfate has declined. Nitrate contributed relatively little to the negative charge in these predominantly forest catchments. Sodium was the dominant cation, contributing approximately half of the positive charge over the period of observation. Both calcium and magnesium concentrations have declined while potassium was low and relatively constant over the whole time period.

**Fig. 5:**
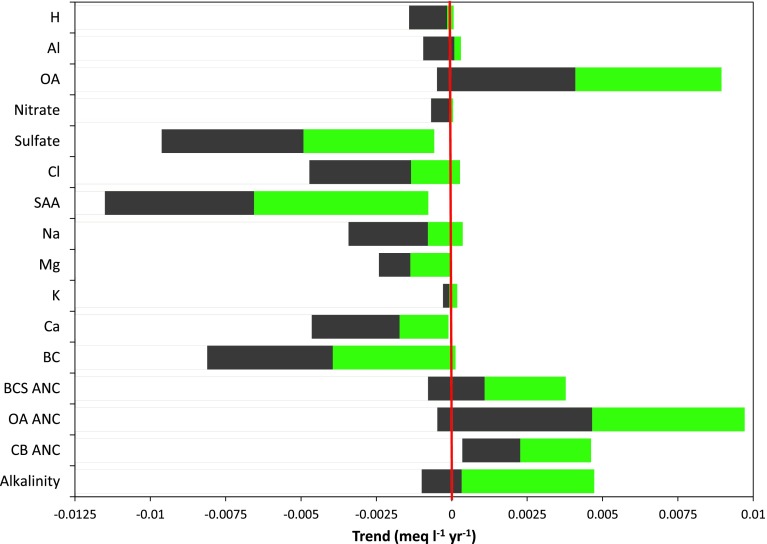
Trend summary for all analyzed parameters. Trends were estimated using the Sen slope. Trends are presented for all parameters from 1987 or 1988–2012, except for alkalinity where trends are reported for 1994–2012. *Grey bars* represent values below the median while *green bars* show values above the median. The break between *grey* and *green* represents the median trend for a determinand. The *red line* represents no change. All trends are expressed in µeq L^−1^ year^−1^

All trends can be summarized and compared in a single figure showing the distribution of Sen slopes (Fig. 5). While [H^+^] is declining in 26 of 30 lakes, most of the declines were very small. On average, total aluminum concentrations increased slightly and with one exception, the concentration of organic acids increased over time. Nitrate concentrations declined slightly in most lakes while sulfate concentrations declined in all lakes. Both increasing and decreasing chloride trends were observed. Strong acid anion, magnesium, and calcium concentrations declined in all lakes. Like chloride, sodium displayed both positive and negative trends. There were small positive and negative trends for potassium. Base cation concentrations declined in all but one lake. Measured alkalinity displayed positive and negative trends over time. With a few exceptions, all estimated ANC parameters (Eqs. 1–3) increased over time in all study lakes. It is noteworthy that CB_ANC increased more rapidly than measured alkalinity and that the two calculated alkalinity parameters which include the effects of strong organic acids showed trend distributions closer to those of measured alkalinity.

**Fig. 6:**
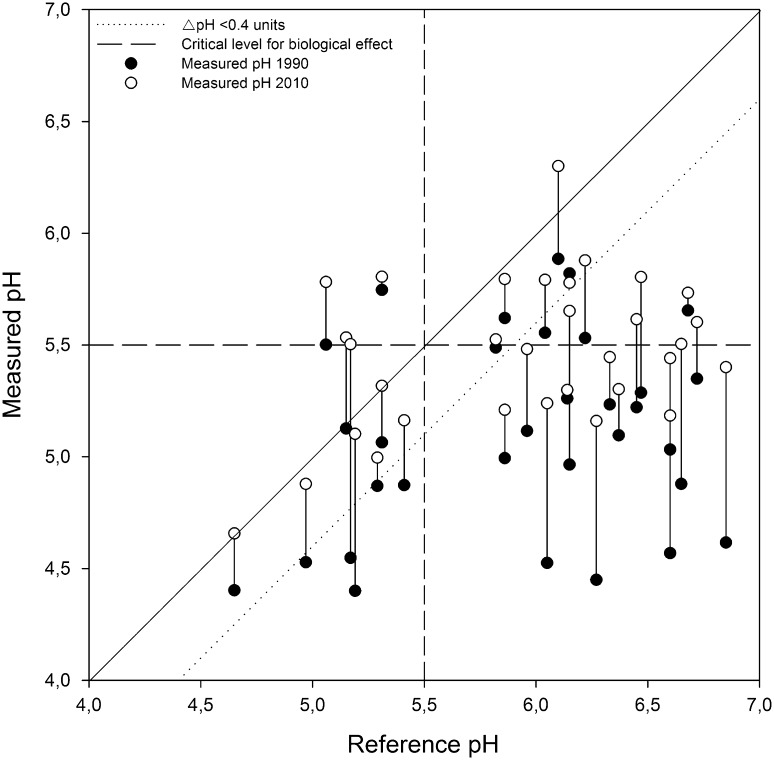
Lake recovery from acidification between 1990 and 2010 as indicated by deviation from reference pH. Reference (MAGIC-estimated) pH is on the* horizontal axis* and measured pH on the* vertical*. Any lake with pH > 0.4 units below the 1:1 line is assumed to have been anthropogenically acidified

Using a conceptual model developed by Valinia et al. (2014), it was possible to compare changes in pH between 1990 and 2010 to reference pH (Fig. 6). The comparison showed that several acidified lakes have recovered during the study period. The solid 1:1 line in Fig. 6 indicates the time when measured pH is equal to reference pH. The dashed line below is the ΔpH criteria of 0.4 pH units from the reference state. All lakes with pH values to the right of this line were assumed to be anthropogenically acidified. In 1990, 22 of 31 lakes were anthropogenically acidified. This figure had declined to 17 of 31 lakes in 2010, 4 of 5 lakes which recovered had reference pH values below 5.5. The conceptual model highlights the fact that some lakes with pH above 5.5 can be considered acidified even though the effects from the change in pH on economically important fish species such as roach were small.

## Discussion

Most of the low-alkalinity lakes in this study are located in the southwest of Sweden, the region that had received the greatest historical acid deposition and suffered the most acidification. These empirical results are consistent with the modeled results of Moldan et al. (2013), which were based on single point in-time measurements. Both empirical and modeled results suggest that many of the sensitive lakes in southwest Sweden still have not completely recovered from the effects of historic acidification. This is further corroborated by the study of Akselsson et al. (2013) showing slow recovery of forest soil solution chemistry in southern Sweden exacerbated by sea-salt episodes.

### Evidence for recovery

Results in this study are consistent with those of Clair et al. (2011) who showed that, despite significant reductions in acid deposition, lakes in eastern Canada only exhibited moderate improvements in alkalinity, increases in DOC, and ongoing declines in calcium. Clair et al. (2011) interpreted these trends as a lack of recovery from acidification. The evidence for recovery from acidification in acid-sensitive Swedish lakes is mixed, and depends in part on definition. If “recovery” is a return to reference conditions, most Swedish lakes have not recovered, but if instead “recovery” is a gradual process of increasing pH and improved biological condition, then lakes in Sweden are recovering. The slight upward long-term trend in measured alkalinity suggests that any recovery is occurring slowly. However, it should be noted that increased concentrations of organic acids may slow any increase in alkalinity. When soil waters with positive alkalinity reach surface waters, organic acids will consume bicarbonate. The fact that the various estimates of ANC are all increasing over time could be interpreted as indicative of recovery. The observed increase in charge balance ANC is a result of strong acid anion concentration declining faster than base cations. This is consistent with the recovery phase of the Cosby et al. (1985) conceptual model of acidification. However, it is not clear how long it will take for soils to recover to pre-acidification levels of base saturation (stage 6) or if the return to a hypothetical pre-acidification state (stage 7) will ever be achieved. Likens and Buso (2012) refer to this alternate trajectory associated with ongoing declines in base cation concentration as “the elusive baseline.” It is not clear that ecosystems ever will recover totally to pre-acidification conditions, and this may have important management implications. For example, it is proposed (Akselsson et al. 2007) that the base cation removal associated with present-day forestry may be enough to prevent recovery of some sensitive lakes. This is important for environmental goal setting since estimates of critical loads, or the amount of acid deposition that will not cause significant harm to ecosystems, have been made using steady-state models (Henriksen and Posch 2001; Bertills and Lövblad 2002). These steady-state models assume that critical load estimates will not change over time. Unfortunately, the on-going decline in base cation concentrations and increasing DOC concentrations mean that critical load estimates will change (Watmough et al. 2005; Erlandsson et al. 2011), supporting the use of dynamic models for critical load estimates. The critical load calculations reported by Sweden to the UNECE in recent years have used MAGIC-forecast base cation concentrations for 2100 instead of a true steady state to account for the observed lack of complete recovery of base saturation (Fölster and Moldan unpublished report).

Overall average pH has increased by approximately 0.5 units between 1987 and 2012, suggesting that most lakes are moving to a reference state. The results presented here suggest that while change in average pH may be sufficient for classifying a lake as not acidified according to the official quality criteria, it may not be enough to sustain biological recovery. Reduced buffering capacity in soils may make lakes more susceptible to episodic re-acidification independent of their change in pH and suggest that acid-sensitive Swedish surface waters are still in the recovery phase (stage 5 or 6) according to the Cosby et al. (1985) conceptual model.

### Estimates of alkalinity

Despite its popularity, the use of CB_ANC as a measure of acidification is problematic. CB_ANC may lack both precision and accuracy. Evans et al. (2001) note that values of CB_ANC near 0 are not precise as they are subject to potentially large uncertainties, resulting from error in the seven measurements needed to estimate strong acid anion concentration and sum of base cations, and the magnitude of estimated CB_ANC relative to that of measurement errors. In the analyses presented here, CB_ANC was not an accurate representation of measured alkalinity, in part because it does not include the effects of organic acidity. CB_ANC was consistently higher than measured alkalinity and showed a more pronounced increase over time. Trends in both Lydersen et al. (2004) and Lawrence et al. (2007) formulations for alkalinity, which included the effects of strong organic acids, were closer to those for measured alkalinity. Given the large discrepancy between measured alkalinity and CB_ANC and the recent increase in surface water DOC, it seems advisable to use either of the DOC-adjusted alkalinity measures. They may provide a more accurate reflection of alkalinity when measurements are not available and incorporate the effects of varying DOC concentrations on surface water acidity status over time.

### Possible forestry effects

More intensive forest harvesting for bioenergy production is becoming increasingly common in Sweden (Laudon et al. 2011). Greater rates of removal of biomass associated with whole-tree harvesting result in greater rates of base cation removal. Modeling studies have suggested that rates of base cation removal associated with both conventional and intensive forest harvesting in Sweden exceed the rate at which they can be replaced through mineral weathering (Akselsson et al. 2007). Modeling studies in Finland suggest that more intensive forest harvesting may lead to surface water re-acidification (Aherne et al. 2012). These results should be contrasted with similar modeling studies in the UK which suggest that more intensive forestry is unlikely to affect the recovery process (Helliwell et al. 2014), and recent Swedish modeling suggests that whole-tree harvesting may not have severe effects on stream water ANC (Zetterberg et al. 2014). While the precautionary principle suggests that the risk of delayed recovery or re-acidification be taken seriously, there is relatively little empirical evidence for soil re-acidification following more intensive forest harvesting (Thiffault et al. 2011). For example, long-term studies at three sites in Sweden indicate that differences in soil water calcium concentrations between whole-tree and stem-only harvest largely had disappeared 32–35 years after harvest. The greatest effects were found at the well-buffered site where the loss of calcium due to intense harvest is less likely to cause acidification. The effects at the two acidic sites were probably not large enough to counterbalance the recovery from acidification (Zetterberg et al. 2013). Furthermore, Klaminder et al. (2011) have noted that weathering rate estimates are not sufficiently precise for reliable use in assessments of forestry-related acidification.

### Open questions

Water chemistry in acid-sensitive Swedish lakes may be moving toward an unprecedented state in which base cation concentrations never return to reference levels. The increase in DOC is consistent with a return to pre-industrial conditions (Cunningham et al. 2011; Valinia et al. 2012). However, reference condition DOC, unusually low base cation concentrations and the potential for increased sea-salt events in a changing climate may make surface waters more prone to episodic acidification. Furthermore, recovery from acidification may be linked to ongoing declines in lake phosphorus concentrations. Nitrogen deposition is still at much higher levels than would have occurred in the past (Moldan et al. 2013) but this is not seen in surface waters, primarily due to strong demand by terrestrial plants.

The shift from mineral to organic acidity is worthy of further investigation. Evans et al. (2008) note that if this shift is associated with a climate change induced increase in DOC instead of being the result of declines in acid deposition, then it can delay the recovery of surface waters from acidification. While modeling studies suggest that the increase in rate of DOC will slow in the future (Holmberg et al. 2014), other studies indicate that DOC has not reached pre-industrial concentrations in many lakes (Cunningham et al. 2011), but this should be corroborated with additional studies.

There has been a significant amount of research directed toward understanding the acid–base properties of natural organic matter in Swedish surface waters (Köhler et al. 2002; Hruška et al. 2003). However, there are still knowledge gaps and more work remains to be done; for example, DOC from wetlands may transport less base cations than DOC from forest sites, and acid functional groups on riparian soil DOC may be counterbalanced by cations instead of protons. Additionally, increasing pH in soils may change DOC solubility and release DOC with a higher site density.

Sodium and chloride are now the most dominant ionic species in many acid-sensitive Swedish lakes. This suggests a significant marine influence on charge balances. Long-term soil solution data from southwest Sweden have shown that recovery from acidification has been delayed by sea-salt deposition (Akselsson et al. 2013). Any increase in storm severity might be expected to have negative effects on the acidification status of both soils and surface waters. However, Lydersen et al. (2002) note that experimental Na addition may reduce Al-related fish mortality. Most of the decline in base cation concentrations is associated with calcium. The current low calcium concentrations in acid-sensitive Swedish lakes may have different effects on fish and zooplankton. Studies in acid-sensitive, low calcium lakes in Canada have suggested that calcium decline may negatively impact zooplankton (Jeziorski et al. 2008). In the most sensitive lakes, calcium concentrations are already below the threshold at which biological effects have been observed (Wærvågen et al. 2002). Norwegian studies have shown that many cladoceran zooplanktons are outcompeted by Holopedium in low calcium lakes (Hessen et al. 1995).

Inorganic Al is one of the most toxic chemical species associated with surface water acidification. Only data on total Al were available in the national monitoring database, but some inferences can be made about possible Al toxicity in acid-sensitive Swedish lakes. While total Al concentrations have not changed appreciably over the period of this study, it is quite likely that toxicity has declined. For example, the recovery from acidification in Norwegian surface waters led to a decrease in toxic (inorganic) aluminum; this decrease was more pronounced in the 1990s than the 2000s (Schartau et al. 2012). The trend toward increasing pH reduces the solubility of inorganic aluminum. Higher DOC concentrations are able to bind more aluminum, thereby reducing the inorganic fraction. Based on modeled data for the period 1990–2006, the inorganic aluminum concentrations decreased in 5 of 10 headwater streams in southwest Sweden (Löfgren et al. 2009). Further modeling studies of the kind presented by Sjöstedt et al. (2010) who studied the relationship between pH, organic matter, iron, and aluminum would be extremely helpful for understanding possible aluminum toxicity in acid-sensitive Swedish lakes and should be complemented by similar assessments to those conducted by Evans et al. (2008) in the UK on the effects of increasing organic acidity on recovery from acidification.

Evans et al. (2010) have recommended the use of z-scores instead of raw data for the comparison of trends between sites. While this approach is very useful for revealing temporal coherence and assessing relative trends with widely different baselines, it can be difficult to relate to ecologically relevant thresholds such as ΔpH or target alkalinities. It may be possible to use observed long-term monitoring data to better inform regional model applications based on single point in-time calibration including the MAGIC simulations described by Moldan et al. (2013). Examination of the patterns in reference and present-day pH shows that lower pH lakes are closer to their reference pH state than higher pH lakes. All lakes with a reference pH < 5.5 have recovered to a non-acidified state while 16 of 21 lakes with a reference pH > 5.5 are still acidified. Many of these acidified lakes have pH values <5.5, suggesting aluminum toxicity may be an on-going issue. From a theoretical point of view, a further recovery in ANC has the largest potential to increase pH in lakes where ANC is close to the inflection point of the S-shaped pH = f(ANC) curve (Ågren and Löfgren 2012). When considering critical levels for biological effects, the 5 acidified lakes with present-day pH > 5.5 may be less in need of remedial measures than those lakes with lower present pH and a reference pH of 5–6 (Fig. 6). However, it should be noted that some waters with a pH between 5.5 and 6 are still very sensitive and further recovery is desirable.

## Conclusion

The overall goal of water management under the Water Framework Directive (WFD; EC 2000) is “good ecological status,” which implies only minor deviations from an undisturbed reference state. For acidification in Sweden, this is based on the ΔpH criteria. This goal creates a practical problem for water management since it is not possible to observe the undisturbed state directly. Thus, management relies on paleolimnological reconstructions or empirical models. The hindcast reconstructions of reference conditions contain uncertainties and are based on contemporary data. Based on the results of this study, two key components can illustrate how decision making in Swedish water management can be improved: (i) We have illustrated the importance of monitoring programs to assess long-term changes in surface waters. Without long-term datasets, water management and classification of reference conditions become ambiguous without the support of monitoring data for surface waters; (ii) Based on the elusive baseline and the acceptance that undisturbed state might be an inadequate criterion for prioritizing water management (Valinia et al. 2014), we can use acid-sensitive lakes as an example to prioritize restoration measures in recovering Swedish surface waters including reintroduction of extirpated species and possibly liming. The acid-sensitive lakes in this study (Fig. 6) illustrate this problem well; most lakes in this study are either acidified or sensitive to acidic episodes that can depress pH and severely affect aquatic biota. The lakes that have recovered above this critical threshold should be prioritized for reintroduction of sensitive fish species since they have the highest chance to establish new populations. For lakes close to the critical limit, reproduction can fail within the years with an acid episode, so introductions might be repeated and the establishment of a new population might take longer. For lakes below the critical level, reintroductions are doomed to fail.

While the results presented here conform to acidification theory, they illustrate the value of long-term monitoring for assessing the effects of pollutant reduction measures, identifying new threats to water quality, and corroborating model results. They can also help to assess the impacts of forest harvesting on lake water chemistry. Most importantly, the long-term monitoring results presented here can be an important tool for informing environmental policy.
